# Patterns of developmental regression and associated clinical characteristics in SLC6A1-related disorder

**DOI:** 10.3389/fnins.2023.1024388

**Published:** 2023-02-21

**Authors:** Sanjana Kalvakuntla, MinJae Lee, Wendy K. Chung, Scott Demarest, Amber Freed, Kyle J. Horning, Terry Jo Bichell, Susan T. Iannaccone, Kimberly Goodspeed

**Affiliations:** ^1^Medical School, University of Texas Southwestern Medical Center, Dallas, TX, United States; ^2^Peter O’Donnell Jr. School of Public Health, University of Texas Southwestern Medical Center, Dallas, TX, United States; ^3^Department of Pediatrics and Medicine, Columbia University, New York, NY, United States; ^4^Department of Pediatrics and Neurology, University of Colorado School of Medicine, Precision Medicine Institute, Children’s Hospital Colorado, Aurora, CO, United States; ^5^SLC6A1 Connect, Dallas, TX, United States; ^6^COMBINEDBrain, Nashville, TN, United States; ^7^Department of Pediatrics, Division of Neurology, University of Texas Southwestern Medical Center, Dallas, TX, United States; ^8^Department of Neurology, University of Texas Southwestern Medical Center, Dallas, TX, United States; ^9^Department of Psychiatry, University of Texas Southwestern Medical Center, Dallas, TX, United States

**Keywords:** *SLC6A1*, neurodevelopmental disorder, epilepsy, developmental regression, autism spectrum disorder

## Abstract

**Introduction:**

SLC6A1-related disorder is a genetic neurodevelopmental disorder that is caused by loss of function variants in the *SLC6A1* gene. Solute Carrier Family 6 Member 1 (*SLC6A1*) gene encodes for gamma-aminobutyric acid (GABA) transporter type 1 (GAT1), which is responsible for reuptake of GABA from the synaptic cleft. Tight regulation of GABA levels plays an important role in brain development by balancing inhibitory and excitatory neuronal signaling. Consequently, individuals with SLC6A1-related disorder can have manifestations such as developmental delay, epilepsy, autism spectrum disorder, and a subset have developmental regression.

**Methods:**

In this study, we identified patterns of developmental regression among a cohort of 24 patients with SLC6A1-related disorder and assessed for clinical characteristics associated with regression. We reviewed medical records of patients with SLC6A1-related disorder and divided subjects into two groups: 1) regression group and 2) control group. We described the patterns of developmental regression including whether there was a trigger prior to the regression, multiple episodes of regression, and whether or not skills were recovered. We assessed the relationship of clinical characteristics among the regression and control groups including demographic factors, seizures, developmental milestone acquisition, gastrointestinal problems, sleep problems, autism spectrum disorder, and behavioral problems.

**Results:**

Individuals with developmental regression had a loss of skills that were previously mastered in developmental domains including speech and language, motor, social, and adaptive skills. The mean age at regression was 2.7 years and most subjects had regression of language or motor skills triggered by seizures, infection, or spontaneously. Although there was no significant difference in clinical characteristics between the two groups, there was a higher prevalence of autism and severe language impairment in the regression group.

**Discussion:**

Future studies of a larger cohort of patients are required to make definitive conclusions. Developmental regression is often a sign of severe neurodevelopmental disability in genetic syndromes, but it is poorly understood in SLC6A1-related disorder. Understanding the patterns of developmental regression and the associated clinical characteristics in this rare disorder will be important to medical management, prognostication, and could impact the design of future clinical trials.

## 1. Introduction

*SLC6A1*-Related disorder (SRD) is caused by loss of function variants in the *SLC6A1* gene (Carvill et al., 2015). Disorders related to this gene have an estimated incidence of 2.65 per 100,000 live births (López-Rivera et al., 2020), and patients commonly present with developmental delay, seizures, and/or autism spectrum disorder (ASD) (Johannesen et al., 2018; Goodspeed et al., 2020). It was first described in 2015 in children with epilepsy with myoclonic atonic seizures (EMAS), developmental delay and generalized epileptiform discharges (Carvill et al., 2015), and is consistently listed among the most frequent genes identified in epilepsy and autism databases (Lindy et al., 2018; Truty et al., 2019; Satterstrom et al., 2020). Solute carrier family 6 member 1 (*SLC6A1*) is a gene expressed in the developing brain that encodes a voltage dependent gamma-aminobutyric acid (GABA) transporter type one (GAT1) (Broer and Gether, 2012). GAT1 is a transmembrane protein that reuptakes the inhibitory neurotransmitter GABA from the synaptic cleft into presynaptic neurons and glia within the central nervous system and plays an important role in brain development by balancing inhibitory and excitatory neuronal signaling (Broer and Gether, 2012; Goodspeed et al., 2020). *SLC6A1* variants found in individuals with SRD consistently demonstrate reduced GAT1 function and impaired trafficking to the cell surface in cell culture (Mattison et al., 2018; Mermer et al., 2021; Trinidad et al., 2022). Disruption of the GAT1 transporter has also been linked to neurological disorders such as attention deficit hyperactivity disorder (ADHD), ASD, intellectual disabilities, epilepsy, and mood disorders (Yuan et al., 2017; Goodspeed et al., 2020).

Although many studies have described the clinical spectrum of SRD, little is known about developmental regression in this disorder. Several cases of developmental regression, especially loss of previously attained language abilities, have been reported. However, there is little information reported on the circumstances, severity, and duration of regression. In a cohort of individuals with EMAS, ten individuals with *SLC6A1* variants were described, four of whom had developmental regression following onset of seizures. In this small cohort, six individuals had autism or autistic traits, including three of the four individuals with developmental regression (Carvill et al., 2015). Language regression was reported in two additional case studies of individuals (Palmer et al., 2016; Islam et al., 2018). In one case, the patient experienced language regression in the setting of increased seizures when a ketogenic diet was held. This patient had slow recovery of language skills after reinitiating the therapeutic diet (Palmer et al., 2016). In the other case, the patient had near normal early language acquisition with at least 50 spontaneous words for labeling and requesting. Language skills were lost at the age of 3-years old when she was also diagnosed with autism spectrum disorder and had onset of seizures and epileptiform discharges on electroencephalogram (Islam et al., 2018). Most reported cases experienced regression before the age of four years. All had a history of developmental delay and seizures, and most had a diagnosis of autism.

In this study, we reviewed the patterns of developmental regression and investigated potential clinical characteristics of regression in our SRD cohort. We defined developmental regression as a loss of skills that were previously mastered including speech and language skills, motor skills, social skills, or adaptive skills. Understanding the patterns and characteristics of developmental regression in SRD is an important component in the development of targeted therapeutic approaches for this rare disorder.

## 2. Materials and methods

### 2.1. Data collection

Medical records of patients with SLC6A1-Related disorder were reviewed from the SLC6A1-Related disorder specialty clinic at the University of Texas Southwestern seen from 2020. Individuals with pathogenic, likely pathogenic, or variants of unknown significance with clinical phenotypes consistent with SLC6A1-Related disorder were included. Demographics, neurological histories, developmental milestones, and frequency of autism spectrum disorder, seizures and semiology, movement problems (ataxia or tremor), gastrointestinal problems (constipation, diarrhea, or feeding problems), sleep problems (problems with sleep initiation or maintenance), and behavioral problems (ADHD, aggression, irritability) were collected. Details regarding development were obtained from guardians, office visits, and medical chart review. Genotype was obtained from review of clinical genetic testing reports. Language impairment is commonly a leading concern for caregivers of children with developmental disability. We defined severe language impairment as having 10 spoken words or fewer. The cohort was divided into individuals with SRD who had a history of developmental regression (Regression Group) and a group with SRD who did not have a history of developmental regression (Control Group). Due to the small sample size and rarity of this disorder, the groups were not matched. We defined developmental regression as loss of a previously obtained motor, language, or social/adaptive skill based on caregiver report and documentation in the medical record. Skills were affirmed to have been established by caregiver report and lost for at least one week. We also characterized whether the individual recovered the previously lost skills. This study was approved by the UT Southwestern Institutional Review Board.

### 2.2. Statistical analysis

Descriptive statistics, including mean and standard deviation for continuous variables, and frequency and percentage (%) for categorical variables, were used to summarize various characteristics. We conducted univariable comparisons of various demographic and clinical characteristics between the Regression and the Control group using a Fisher’s Exact test for categorical variables (e.g., prevalence of autism, seizures, gastrointestinal problems, sleep problems, and behavioral problems) and Student’s *t*-test for continuous variables including age of onset of seizures, age at first clinical concern, age at attaining developmental skills (sitting, walking, babbling, first-word, phrased speech). We checked underlying assumptions, including data normality, when conducting statistical tests. All statistical analyses were run on SAS 9.4 (SAS Institute Inc., Cary, NC, USA) at a 0.05 significance level.

## 3. Results

### 3.1. Developmental milestones in regression vs. control groups

**FIGURE 1:**
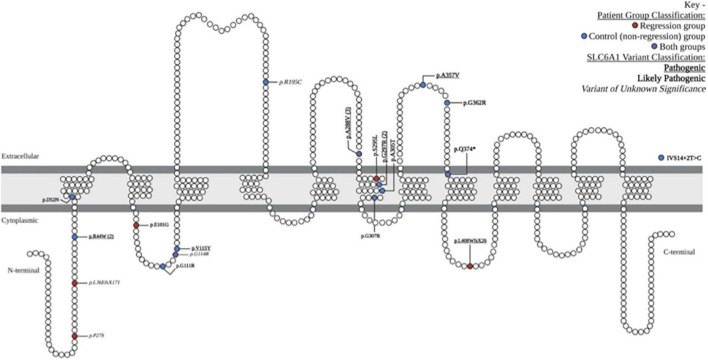
Locations and characteristics of patient-reported variants along GAT-1 protein: schematic demonstration of patient-reported variants along the *SLC6A1* gene, causing the associated symptoms of SLC6A1-related disorder. The 19 unique variants are depicted in their relative location along the gamma-aminobutyric acid (GABA) transporter type 1 protein. One intronic variant is depicted outside of the GAT1 schematic (IVS14 + 2T > C). Variants associated with the regression group are depicted in red, control (non-regression) group are in blue, and those seen in both groups are depicted in purple. Pathogenic variants are denoted with bold underline font, likely pathogenic with bold italic, and variants of unknown significance in *ITALIC.* Schematic adapted from prior publications of SLC6A1-related disorder (Johannesen et al., 2018; Mermer et al., 2021; Kahen et al., 2022).

We reviewed medical records of 24 individuals with SRD. Eight of the 24 participants had a history of developmental regression (Table 1). There were equal numbers of males and females (8:8) in the control group, but a higher number of females (5:3) in the Regression group. There were 19 unique *SLC6A1* variants within the cohort, 2 of which were seen in both the Regression and Control Groups (Figure 1). The mean age at baseline assessment in the Regression Group was 6.8 years ± 3.3 years and was 6.6 years ± 6.2 years in the Control Group. All participants came to medical attention with hypotonia and delayed milestones in infancy or with seizure onset as a toddler. The average age of initial concern in the regression group was 12 months (range: 3–24 months) and was 6.7 months (range: birth to 24 months) in the Control group. Among the regression group, the first concern was delayed motor milestones in 5/8, delayed language skills in 2/8, and seizures in 1/8 of the participants. Among the control group, the first concern was delayed motor milestones in 14/16, delayed language skills in 1/16, and seizures in 1/16 of the participants. Participants in the regression group (*n* = 8) had a mean age of sitting of 9.5 months (range: 5–13 months) and a mean age of walking of 21.9 months (range: 12–33 months). Six of the 8 attained their first word and 3 of 8 had phrased speech by the time of evaluation. Among those with expressive speech, they attained their first word at a mean age of 20.7 months (range: 10–36 months) and phrased speech at a mean age of 37.2 months (range: 24–54 months). Participants in the control group (*n* = 16) sat independently at a mean age of 9.1 months (range: 6–12 months) and walked at a mean age of 17.9 months (range: 11–24 months). In the Control group, 15/16 attained their first word (mean age of 26.4 months, range: 10–52 months), and 9/16 had phrased speech at a mean of 33.9 months (range: 23–48 months).

**TABLE 1:** Demographics and clinical characteristics.

Variable	All *n* = 24	Control *n* = 16 (66.67%)	Regression *n* = 8 (33.33%)	*p*-value*
Male gender, *n* (%)	11 (45.8)	8 (50.0)	3 (37.5)	0.68
Age (years), mean (SD)	6.47 (5.3)	6.64 (6.2)	6.14 (3.1)	0.83
*SLC6A1* Variant type, *n* (%)				
Missense	19 (79.2)	13 (81.2)	6 (75.0)	0.99
Nonsense	5 (20.8)	3 (18.7)	2 (25.0)	0.99
**Clinical characteristics, *n* (%)**				
Severe language impairment	9 (37.5)	5 (31.2)	4 (50.0)	0.41
Autism spectrum disorder	11 (45.8)	5 (31.2)	6 (75.0)	0.08
Epilepsy	22 (91.7)	14 (87.5)	8 (100.0)	0.54
Drug resistant epilepsy, *n* (%)	13 (54.2)	9 (56.2)	4 (50.0)	0.99
Absence seizures, *n* (%)	22 (91.7)	14 (87.5)	8 (100.0)	0.54
Atonic seizures, *n* (%)	14 (58.3)	9 (56.2)	5 (62.5)	0.99
Myoclonic seizures, *n* (%)	10 (41.7)	6 (37.5)	4 (50.0)	0.67
Movement problem	21 (87.5)	14 (87.5)	7 (87.5)	0.99
Gastrointestinal problem	20 (83.3)	13 (81.2)	7 (87.5)	0.99
Behavior problem	21 (87.5)	14 (87.5)	7 (87.5)	0.99
Sleep problem, *n* (%)	16 (66.7)	11 (68.7)	5 (62.5)	0.99
IRDA, *n* (%)^a^	15 (100.0)	9 (100.0)	6 (100.0)	N/A
NICU, *n* (%)^b^	7 (36.8)	6 (42.9)	1 (20.0)	0.60
Age at ASD diagnosis (months), mean (SD)^c^	45.09 (23.3)	46.00 (20.5)	44.33 (27.4)	0.91
Age at seizures absence (months), mean (SD)^d^	21.55 (14.4)	21.86 (16.2)	20.83 (10.3)	0.89
Age at seizures atonic (months), mean (SD)^e^	23.50 (8.0)	23.60 (7.8)	23.25 (9.9)	0.94
Age at first concern (months), mean (SD)	8.21 (7.1)	6.69 (6.0)	11.25 (8.6)	0.14
**Age (months), mean (SD) at acquisition of^f^**				
Sitting	9.23 (2.4)	9.07 (2.3)	9.50 (2.8)	0.70
Walking	19.14 (5.9)	17.46 (4.3)	21.88 (7.2)	0.09
Babbling	14.26 (8.5)	15.50 (9.5)	12.14 (6.6)	0.42
First-word	24.80 (11.5)	26.57 (12.1)	20.67 (9.5)	0.30
Phrased speech	35.07 (11.6)	33.89 (9.7)	37.20 (15.5)	0.63

Summary of the gender, age, genotype, and clinical characteristics of the regression group and control group. There is no significant difference between any demographic or clinical characteristics Fisher’s Exact (categorical variables) or student’s t-test (continuous variable).

*Fisher’s Exact test for categorical variables; *t*-test for continuous variables, SD, standard deviation.

^a^Intermittent rhythmic delta (IRDA) *n* = 9 control, *n* = 6 regression.

^b^*n* = 14 control, *n* = 5 regression.

^c^*n* = 5 control, *n* = 6 regression.

^d^*n* = 14 control, *n* = 6 regression.

^e^*n* = 10 control, *n* = 4 regression.

^f^*n* = 2–10 missing.

### 3.2. Overview of developmental regression

Eight of our 24 participants had a history of developmental regression (Table 2). Six of the eight had one or more episodes of regression with subsequent recovery of the previously lost skills. One participant had regression associated with an infection, 1 was associated with a life stressor, 1 was associated with seizures, and 5 had no identified trigger. Six of the eight had recovery of previously lost skills after the period(s) of regression, and two had no meaningful recovery of lost skills (language). The mean age at regression was 32.5 months (range 12–60 months). Four of the 8 had a loss of motor skills, six of the eight had a loss of language skills, and three of the eight had a loss of social/adaptive skills. Five of the eight had a loss of skills in more than one developmental domain.

**TABLE 2:** Overview of regression group.

ID	Gender	*SLC6A1* genotype	Variant classification	Inheritance	Regression history					Other clinical features		
					**Age of onset**	**Type**	**Triggers**	**Recovery**	**Episodic**	**Autism**	**Epilepsy**	**Types of seizures**
**Regression group**												
1	Male	c.884 C > T p.S295L	Likely pathogenic	*De novo*	3y 3m	Mild language	Not identified	Yes	Yes	Yes	Yes	Absence
2	Female	c.863 C > T p.A288V	Pathogenic	*De novo*	1y 10m	Severe motor	Infection	Yes	Yes	Yes	Yes	Atonic
3	Male	c.1222delC p.L408WfsX26	Pathogenic	N/A	2y	Severe motor	Not identified	Yes	Yes	No	Yes	Atonic, myoclonic
4	Female	c.104dupA p.L36EfsX171	Pathogenic	N/A	5y	Severe language; moderate motor	Cross-country move	No; yes	No	Yes	Yes	Atonic, myoclonic
5	Female	c.340 G > A p.G114R	VUS	N/A	1y 3m	Severe language; mild daily living skills	Not identified	Yes	Yes	Yes	Yes	Absence, myoclonic
6	Male	c.863 C > T p.A288V	Pathogenic	*De novo*	1y	Sever language and social skills	Not identified	No	No	Yes	Yes	Atonic
7	Female	c.79 C > T p.P27S	VUS	N/A	4y 4m	Mild language; moderate motor	Seizures	Partial	No	Yes	Yes	Atonic, focal motor
8	Female	c.302 A > G p.E101G	Likely pathogenic	*De novo*	3y	Severe language; social skills	Not identified	No	No	Yes	Yes	Atonic, absence, myoclonic
**Control/Non-regression group**												
9	Male	c.863 C > T p.A288V	Pathogenic	Maternal						Yes	Yes	Absence
10	Male	c.130 C > T p.R44W	Pathogenic	*De Novo*						Yes	Yes	Atypical absence, myoclonic, GTC
11	Male	c.154G > A p.D52N	Likely pathogenic	*De Novo*						Yes	Yes	Absence, atonic
12	Male	c. 1084 G > A p.G362R	Likely pathogenic	*De Novo*						No	Yes	Atonic, myoclonic, Absence
13	Male	c.339delA p.V115Y	Pathogenic	Unknown						No	Yes	Absence, atonic
14	Male	c.331 G > A p.G111R	Likely pathogenic	*De Novo*						Yes	Yes	Absence
15	Female	c. 130C > T p.R44W	Likely pathogenic	*De Novo*						No	No	
16	Female	c.889G > A p.G297R	Pathogenic	*De Novo*						Yes	Yes	Absence, atonic
17	Female	c.1527 + 2T > C IVS14 + 2T > C	Pathogenic	*De Novo*						Yes	Yes	Atonic, myoclonic
18	Female	c.583C > T p.R195C	VUS	Unknown						No	Yes	Myoclonic
19	Female	c. 889 G > A p.G297R	Pathogenic	*De Novo*						No	Yes	Atonic, absence
20	Female	c.1070 C > T p.A357V	Pathogenic	Paternal						No	Yes	Absence, atonic
21	Male	c.913 G > A p.A305T	Likely pathogenic	Unknown						Yes	Yes	Absence, atonic, myoclonic
22	Female	c.340 G > A p. G114R	VUS	Unknown						No	No	
23	Male	c.919G > A p.G307R	Likely pathogenic	*De Novo*						No	Yes	Absence, myoclonic, Atonic, GTC
24	Female	c. 1120 C > T p.Q374*	Likely pathogenic	*De Novo*						No	Yes	Absence

Summary of demographics, genotype, characteristics of developmental regression and clinical characteristics including autism spectrum disorder, epilepsy, and seizure semiology (GTC − generalized tonic clonic).

### 3.3. Clinical characteristics associated with developmental regression

Table 1 shows comparisons of various characteristics between groups (regression and control). There was no statistical difference between the regression vs. control groups in prevalence of behavior problems or movement disorder. Though not statistically significant, the regression group had higher prevalence of seizures (100% vs. 87.5%) and gastrointestinal problems (87.5% vs. 81.3%), and lower prevalence of missense variants (75% vs. 81.3%), drug resistant epilepsy (50% vs. 56.3%), sleep problems (62.5% vs. 68.8%), and neonatal intensive care unit admissions (20% vs. 42.9%) compared to the controls. Although there were higher rates of each seizure type in the regression group compared to controls including absence (100% vs. 87.5%), atonic (62.5% vs. 56.3%), and myoclonic (50% vs. 37.5%), these differences did not reach statistical significance. All individuals in the study had intermittent rhythmic delta activity (IRDA) on EEG. There was a higher prevalence of ASD in the regression group compared to the control group (75% vs. 31.3%), but this did not reach statistical significance (*p* = 0.08). The mean age at ASD diagnosis was similar between both groups (control 3.8 years, regression 3.6 years, *p* = 0.91). There was no statistically significant difference between regression and control groups in the mean age at first clinical concern (11.3 vs. 6.7 months, *p* = 0.14), or mean age at onset of seizures. There was also no statistically significant difference in the mean age at acquisition of sitting, walking, babbling, first-word, or phrased speech between the two groups. Among the regression group, four of the eight participants (50%) had severe language impairment. In the control group, 5 of the 16 (31%) had severe language impairment, but this difference was not statistically significant (*p* = 0.41).

## 4. Discussion

Developmental regression was common in this study, affecting approximately one-third of our cohort. Though this was a small study and no clinical characteristics reached statistical significance, there were some trends identified that warrant future investigation with a larger group. All individuals in this study had a history of developmental delay, but individuals with a history of regression tended to reach their developmental milestones at a later age than the controls. Additionally, the age at which children came to medical attention was older in the regression group and most often related to the onset of seizure-like activity. It was also notable that there was a higher prevalence of severe language impairments and autism spectrum disorder in the regression group. Although we did not identify a significant relationship between clinical features and developmental regression, there was a higher prevalence of ASD in the regression group. ASD is a neurodevelopmental disorder that is known to be associated with developmental regression in fewer than one third and occurs at a mean age of 19 months of individuals (Tan et al., 2021). This warrants further exploration to understand if a diagnosis of ASD is a risk factor for regression in a larger cohort of patients with SRD.

The study also explored whether the regression group had a common identifiable trigger for the regression. There was no identifiable trigger of developmental regression in the majority of patients, but one child in the regression group had an infection around the time of their developmental regression. This is not surprising as many neurological disorders tend to worsen when patients have an infection, but this trigger was less prevalent than anticipated. One child also had a life stressor as a theorized potential trigger. It is notable, however, that these triggers are theoretical and based on both physician and guardian observation as it is still unclear whether the regression associated with SRD is spontaneous or induced. There was not a clear worsening of seizure frequency that correlated with the periods of regression or infections. Further, even those individuals with good seizure control still experienced developmental regression and the individual with the most severe seizure disorder (daily seizures on adequate dosing of three anti-seizure medications) has never had a period of developmental regression. In this study, it does not appear that seizure severity, age at seizure onset, or seizure semiology correlates with developmental regression. Further, while some individuals with SRD will meet criteria for epileptic encephalopathy, such as epilepsy with myoclonic atonic seizures (EMAtS) or Lennox-Gastaut syndrome (LGS), many individuals with SRD have a well-organized background rhythm with interictal abnormalities. In our cohort, only one individual meets criteria for LGS and had no history of developmental regression.

This study is limited by the small sample size, but it is the largest study to date to investigate developmental regression in SRD. Published case reports suggested a predominance of females, but there was an even distribution of gender between both groups in our cohort. There were several patterns of developmental regression reported in our cohort and regression of language skills was the most prevalent. Individuals either had a single period of regression without recovery, a single period of regression with recovery, or multiple episodes of regression with recovery and subsequent developmental progression. The sample was too small to adequately explore clinical differences between these three observed patterns. Additionally, with the cross-sectional design and relatively young age of this cohort, it is possible that a patient in the control group could still go on to have regression or that patients with a single period of regression could go on to have additional episodes of regression. Within our control group, there were 9 participants under the age of 5 years, 5 of whom were under the age of 3 years old. A longitudinal study is needed to adequately define regression patterns and clinical characteristics associated with regression.

## 5. Conclusion

Developmental regression is a prevalent feature of SRD, affecting one-third of our cohort. We identified three patterns of developmental regression: (1) single episode without recovery, (2) single episode with recovery, and (3) multiple episodes with recovery. While ASD and speech impairments were more prevalent in the regression group, this finding did not reach statistical significance and these clinical features were also seen in the control group. No other clinical characteristics were clearly associated with developmental regression. Future studies should investigate SRD related developmental regression among a larger cohort in order to better define the full clinical spectrum and characteristics of this disease.

## Data availability statement

The raw data supporting the conclusions of this article will be made available by the authors, without undue reservation.

## Ethics statement

The studies involving human participants were reviewed and approved by the University of Texas Southwestern Institutional Review Board. Written informed consent to participate in this study was provided by the participants’ legal guardian/next of kin.

## Author contributions

SK and KG contributed to the study design, data collection and analysis, and drafting of the manuscript. ML contributed to the study design, data analysis, and drafting of the manuscript. WC, SD, AF, KH, TB, and SI contributed to the data analysis and significant manuscript revisions. All authors contributed to the article and approved the submitted version.
